# Metabolic dysfunction-associated steatotic liver disease and bone mineral density in type 2 diabetes mellitus: insights from a single-center cross-sectional study in North India

**DOI:** 10.3389/fendo.2026.1758966

**Published:** 2026-03-02

**Authors:** Dogga Sudhakar, Sumit Rajotiya, Sourav Debnath, Nishant Jain, Monal Sherekar, Shivang Mishra, Anurag Kumar Singh, Mahaveer Singh, Avinash Munshi, Deepak Nathiya, Balvir Singh Tomar

**Affiliations:** 1 Department of Endocrinology, National Institute of Medical Sciences and Research Hospital, Nims University Rajasthan, Jaipur, India; 2 Department of Pharmacy Practice, Nims Institute of Pharmacy, Nims University Rajasthan, Jaipur, India; 3 Family Medicine Consultant, Connect and Heal Primary Care Pvt Ltd., Bengaluru, Karnatak, India; 4 Department of Radiology, National Institute of Medical Sciences and Research Hospital, Nims University Rajasthan, Jaipur, India; 5 Institute of Gastroenterology, Hepatology & Transplant, Nims University Rajasthan, Jaipur, India

**Keywords:** bone mineral density, fibrosis-4 index, India, metabolic dysfunction-associated steatotic liver disease, type 2 diabetes mellitus

## Abstract

**Introduction:**

Metabolic dysfunction-associated steatotic liver disease (MASLD), recently redefined from non-alcoholic fatty liver disease, commonly coexists with type 2 diabetes mellitus and may adversely affect skeletal health through inflammation and insulin resistance. However, research on site-specific bone mineral density (BMD) effects in South Asian populations, particularly Indians, remains limited. This study aimed to evaluate the association between MASLD and BMD at different skeletal sites among adults with type 2 diabetes mellitus in North India.

**Methods:**

This cross-sectional study included 100 adults (50 with T2DM and 50 controls) at a tertiary care center in Jaipur, India. MASLD was evaluated using the fibrosis-4 (FIB-4) index, hepato-renal index, and two-dimensional shear wave elastography. BMD at lumbar spine (L1-L4), left total hip (proximal femur), and left forearm was assessed by dual-energy X-ray absorptiometry. Associations between MASLD parameters and BMD were analyzed using multivariable linear regression, adjusting for age, sex, and smoking status.

**Results:**

T2DM patients showed significantly lower lumbar spine (L1-L4) BMD (0.93 vs. 0.98 g/cm^2^; P = 0.026) and T-scores (-1.33 vs. -0.72; P = 0.004), especially in males ≥50 years. No statistically significant differences were observed in proximal femur and forearm BMD between groups. FIB-4 scores were higher (P = 0.004), with 60% at intermediate-to-high fibrosis risk versus 28% of controls; advanced fibrosis occurred in 10% vs. 2%. Hepatic steatosis positively associated with hip BMD (β=0.738; P = 0.022).

**Conclusion:**

This study establishes significant, site-specific associations between MASLD and reduced bone mineral density in type 2 diabetes mellitus patients, particularly affecting trabecular-rich skeletal sites. The complex relationship between hepatic steatosis and skeletal health highlights the multisystemic nature of metabolic dysfunction. These findings support integrating bone health assessments and non-invasive hepatic fibrosis screening into routine diabetes care to improve early risk stratification.

## 1 Introduction

Metabolic dysfunction-associated steatotic liver disease (MASLD) is the most prevalent chronic liver condition worldwide, paralleling the rise in obesity, type 2 diabetes mellitus (T2DM), and sedentary lifestyles (1). By 2021, approximately 1.27 billion adults were affected, representing a 24% increase since 1990, and current projections suggest that MASLD may affect more than half of the world’s adult population by 2040 (2).

In India, the burden is substantial. A meta-analysis reported a prevalence of 38.6% among adults, while community-based FibroScan surveys found hepatic steatosis in 68% and significant fibrosis in 34% of 13,750 participants (3, 4). T2DM confers a two- to three-fold higher risk of advanced fibrosis, and nearly 70% of Indians adults with diabetes are affected by MASLD (5).

In June 2023, an international Delphi consensus redefined non-alcoholic fatty liver disease (NAFLD) as MASLD. Diagnosis requires ≥5% hepatic steatosis on imaging or histology plus at least one cardiometabolic risk factor such as overweight/obesity, T2DM/prediabetes, hypertension, dyslipidemia, or metabolic syndrome after excluding excess alcohol and viral hepatitis (6–9).

MASLD also exerts systemic effects, including on bone health. Chronic inflammation (IL-6, TNF-alpha), insulin resistance, and hepatokine dysregulation (FGF-21, fetuin-A, osteopontin) disrupt osteoblast-osteoclast balance, promoting resorption (10). Meta-analyses involving >30,000 adults show reduced lumbar and femoral bone mineral density (BMD), with increased osteoporosis prevalence (17–33%) and fragility fractures (~35%), especially in Asian men and postmenopausal women (11). However, other studies have reported preserved or even higher BMD in MASLD, especially in individuals with obesity or T2DM, possibly due to mechanical loading and hyperinsulinemia, highlighting substantial heterogeneity in skeletal outcomes (12).

Importantly, T2DM itself exerts complex and often paradoxical effects on bone health, characterized by normal or increased BMD but disproportionately elevated fracture risk due to impaired bone quality, microarchitecture, and material properties. The coexistence of T2DM and MASLD may therefore have additive or synergistic effects on skeletal integrity that are not adequately captured by BMD alone (13).

Despite the high prevalence of MASLD in the Indian population, there is limited evidence linking MASLD severity with bone health using non-invasive fibrosis assessment tools among individuals with T2DM. Moreover, most available studies have not specifically examined this association in individuals with T2DM, in whom metabolic and skeletal interactions may differ (14). The present study aimed to investigate the association between MASLD and BMD in adults with T2DM in North India using comprehensive non-invasive tools.

## 2 Methods

### 2.1 Study subjects

This single-center, cross-sectional study was conducted at a tertiary teaching care hospital in North India. Ethical clearance was obtained from the Institutional Ethics Committee (IEC). All participants provided written informed consent prior to enrollment. All procedures performed in this study complied with the ethical standards of the Indian Council of Medical Research (ICMR) and the Declaration of Helsinki (1975) and followed the STROBE guidelines for observational studies.

**Figure 1:**
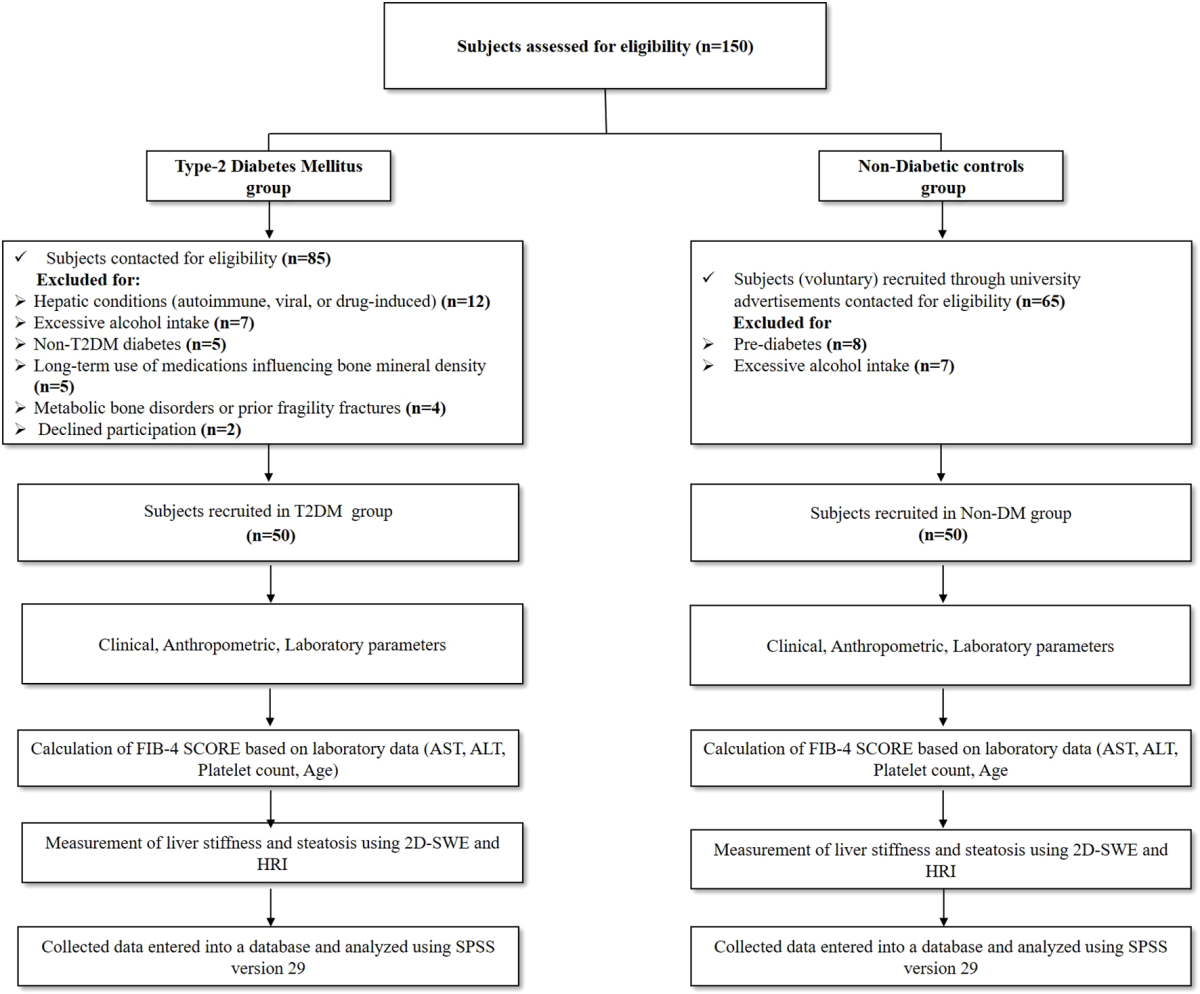
Flow chart of the study participants between T2DM patients and non-diabetic controls.

A total of 100 adults (>18 years) were enrolled, including 50 patients with established type 2 diabetes mellitus (T2DM) recruited from the Endocrinology outpatient department and 50 healthy volunteers (non-diabetic controls with HbA1c <5.6%) recruited through university advertisements. Participants underwent evaluation for MASLD using hepato-renal index (HRI), FIB-4 score, and shear-wave elastography. MASLD status and severity were determined based on these non-invasive assessments. Exclusion criteria included known chronic liver diseases (including viral hepatitis, autoimmune hepatitis, biliary disease, or cirrhosis), chronic kidney disease, prior fragility fractures, prolonged immobilization, or diagnosed metabolic bone disorders such as osteopenia, osteoporosis, osteomalacia, or Paget’s disease. Participants receiving medications known to affect bone or liver metabolism (including glucocorticoids, bisphosphonates, thiazolidinediones, or steatogenic drugs), those with significant alcohol consumption (>21 standard drinks/week for men and >14 drinks/week for women), and pregnant or lactating women were also excluded. (**Figure 1**).

### 2.2 Clinical, anthropometric, and biochemical evaluation

Upon recruitment, all participants underwent structured interviews and physical examinations, with data recorded in pre-designed case record forms. Demographic details (age, gender, address, marital status) and lifestyle factors (smoking, alcohol use, physical activity, dietary patterns, education, occupation, family history) were collected, along with presenting symptoms, medical history, and comorbidities including hypertension and dyslipidemia. Anthropometric assessments included height, weight, waist, and hip circumferences using standardized methods. Body mass index (BMI) was calculated as weight (kg)/height^2^ (m^2^) and classified using Asia-Pacific cut-offs: normal (18.5–22.9), overweight (23.0–24.9), obese class I (25.0–29.9), and obese class II (≥30.0 kg/m^2^) (15).

Biochemical analyses were performed in a certified laboratory using standardized protocols. Fasting venous blood samples (≥8 hours) were collected. Complete blood counts, including hemoglobin, leukocyte, and platelet counts, were measured with an automated hematology analyzer (Mindray BC-6000, Shenzhen, China). Serum chemistry was assessed on the Vitros 5600 Integrated System (Ortho Clinical Diagnostics, USA). Renal function (creatinine, urea) was evaluated by enzymatic colorimetry, while electrolytes were estimated using ion-selective electrodes. Liver function tests included albumin, bilirubin, alkaline phosphatase, and aminotransferases. Lipid profile, vitamin D [25(OH)D], and glycated hemoglobin (HbA1c) were measured using validated enzymatic, immunoassay, and HPLC methods, respectively.

Additionally, the FIB-4 index was calculated using the formula: FIB-4 index = age (years) × AST (U/L)/(PLT (×109/L) × √ALT (U/L). Scores were interpreted as <1.3 (low risk), 1.3–2.67 (intermediate risk), and >2.67 (high risk of advanced fibrosis) (16).

### 2.3 Radiological investigations

#### 2.3.1 Assessment of bone mineral density

Bone mineral density (BMD) was assessed using dual-energy X-ray absorptiometry (DXA), the reference standard for evaluation of skeletal health. All participants were scanned using the same DXA system Horizon Wi densitometer (Hologic Inc., Marlborough, Massachusetts, USA) operating in the standard adult acquisition mode. Participants were instructed to discontinue calcium supplementation for at least 72 hours prior to scanning and to wear loose, non-metallic clothing. BMD measurements were obtained at the lumbar spine (L1–L4), total left hip, and left forearm, in accordance with International Society for Clinical Densitometry (ISCD) guidelines.

T-scores were calculated using the manufacturer-provided NHANES reference population and interpreted as normal (> −1.0), osteopenia (−1.0 to −2.5), and osteoporosis (≤ −2.5); in individuals with diabetes, a T-score threshold of −2.0 was considered equivalent to osteoporosis (17–19). Regions of interest were automatically delineated by the software and manually adjusted when required to ensure accuracy. TBS analysis was not available for this study, as the DXA system used did not have the licensed TBS software installed at the time of data acquisition. The DXA system underwent daily calibration, regular preventive maintenance by certified engineers, and the least significant change (LSC) was set at 1.2.

#### 2.3.2 Assessment of hepatic steatosis and fibrosis

Hepatic steatosis and fibrosis were evaluated using B-mode ultrasonography combined with quantitative measurements obtained via the Hepato-Renal Index (HRI) and two-dimensional Shear Wave Elastography (2D-SWE), performed on the *Mindray Resona I9* system by experienced radiologists. The HRI was calculated as the ratio of hepatic to renal cortical echogenicity, with steatosis classified as mild (1.05–1.24), moderate (1.25–1.64), or severe (≥1.65) (20). Liver stiffness was measured in kilopascals (kPa) using SWE and interpreted according to standard cut-offs: F0–F1 (no or mild fibrosis) ≤5.0 kPa, F2 (significant fibrosis) 5.1–8.9 kPa, F3 (advanced fibrosis) 9–13 kPa, and F4 (cirrhosis) >13 kPa.

#### 2.3.3 Sample size estimation and study power

As this was a cross-sectional observational study, the sample size was estimated using Cochran’s formula for prevalence-based studies: *n = Z^2^p(1−p)/d^2^*, where *Z* represents the standard normal deviate at a 95% confidence level (1.96), *p* the expected proportion, and *d* the margin of error. A conservative expected proportion of 50% (*p* = 0.5) was assumed to maximize the required sample size, with a margin of error of 10%. Based on these assumptions, the minimum required sample size was approximately 96 participants. To account for an anticipated 5% non-response rate, the sample size was adjusted to approximately 101 participants. A total of 100 participants were ultimately recruited, with equal allocation between the two groups (50 per group), closely approximating the adjusted estimate. Given the observational and exploratory nature of the study, a separate *a priori* power calculation was not performed; however, a *post-hoc* assessment based on the observed effect size of the primary outcome indicated adequate statistical power (≥80%) for the planned multivariate analyses.

#### 2.3.4 Statistical analysis

Statistical analysis for this study was performed using Microsoft Excel (Version 2019; Microsoft Corporation, Redmond, WA, USA) and IBM Statistical Package for the Social Sciences (SPSS) Statistics (Version 29.0; IBM Corp., Armonk, NY, USA). The normality of continuous variables was assessed using the Kolmogorov–Smirnov test. Comparisons between groups were performed using the independent samples Student’s *t*-test for continuous variables and the Chi-square test for categorical variables.

Correlation analysis between bone mineral density and relevant clinical and biochemical variables was conducted using the Pearson correlation coefficient. To evaluate the association between metabolic dysfunction–associated steatotic liver disease (MASLD) and bone mineral density, linear regression analysis was performed with the bone mineral density *T*-score as the dependent variable. Independent predictors of bone mineral density were identified using multivariate linear regression models while adjusting for relevant clinical covariates. Results are presented as unstandardized beta coefficients with their corresponding 95% confidence intervals. All statistical tests were two-tailed, and a *p*-value < 0.05 was considered statistically significant. Graphical representations were generated utilizing GraphPad Prism version 9.0.

## 3 Results

### 3.1 Participant recruitment and study flow

From December 2024 to May 2025, a total of 150 subjects were assessed for eligibility. In the T2DM group, 85 individuals were screened, of whom 35 were excluded (12 due to hepatic conditions, 7 due to excessive alcohol intake, 5 with non-T2DM diabetes, 5 receiving long-term medications affecting bone mineral density, 4 with bone-affecting conditions, and 2 who declined participation). Ultimately, 50 T2DM patients were enrolled.

In the non-diabetic control group, 65 volunteers were screened; 15 were excluded (8 with pre-diabetes and 7 with excessive alcohol intake), resulting in 50 non-diabetic controls. Thus, 100 participants (50 T2DM and 50 non-diabetic) were included in the final analysis (**Figure 1****).**

### 3.2 Baseline demographic and clinical characteristics

The mean age was comparable between the two groups (T2DM: 51.38 ± 7.96 years vs. non-diabetic controls: 49.30 ± 11.97 years; p = 0.308). There were no statistically significant differences between groups with respect to sex distribution, residence, smoking status, body mass index (BMI), or BMI category distribution (**Table 1****).**

**Table 1:** Baseline socio-demographic characteristics of the study participants.

Variables	T2DM (n=50)	Non-DM (n=50)	P-value
**Age, mean ± SD**	51.38 ± 7.96	49.30 ± 11.97	0.308
Gender, n (%)			
Male	37 (74%)	26 (52%)	0.23
Female	13 (26%)	24 (48%)	
Residence, n (%)			
Rural	33 (66%)	26 (52%)	0.15
Urban	17 (34%)	24 (48%)	
Smoking status, n (%)			
Yes	10 (20%)	4 (8%)	0.084
No	40 (80%)	46 (92%)	
**BMI, mean ± SD**	27.64 ± 4.43	28.17 ± 4.94	0.576
BMI categories, n (%)			
Normal	4 (8%)	6 (12%)	0.551
Overweight	10 (20%)	8 (16%)	
Obese Class 1	25 (50%)	20 (40%)	
Obese Class 2	11 (22%)	16 (32%)	

All the continuous variables are presented in mean ± standard deviation (SD) and categorical variables are presented in number (%). p-value was significant at less than 0.05. Significant values are marked bold.

### 3.3 Laboratory and biochemical parameters

Participants with T2DM demonstrated significantly higher pulse rate (p = 0.035), systolic blood pressure (p < 0.01), diastolic blood pressure (p = 0.028), hemoglobin (p = 0.029), and packed cell volume (p = 0.007) compared to non-diabetic controls. Glycated hemoglobin (HbA1c) levels were markedly elevated in the T2DM group (8.50 ± 1.42 vs. 5.20 ± 0.22; p < 0.01), reflecting the inclusion criteria.

Electrolyte analysis revealed significantly higher serum potassium levels in the T2DM group (p < 0.01), while serum sodium levels were comparable. Liver function tests showed higher direct bilirubin (p = 0.005), serum glutamic-oxaloacetic transaminase (SGOT; p = 0.047), and alkaline phosphatase (ALP; p < 0.01) among diabetic participants. Lipid profile analysis demonstrated higher total cholesterol levels in the T2DM group (p = 0.012), whereas high-density lipoprotein, low-density lipoprotein, and triglyceride levels did not differ significantly. Renal function parameters, serum albumin, globulin, and vitamin D levels were comparable between the two groups (**Table 2****).**

**Table 2:** Biochemical and hematological profile of study participants in T2DM and non- diabetic controls.

Variables, mean ± SD	T2DM (n=50)	Non- DM (n=50)	P-value
Pulse (bpm)	86.64 ± 11.88	82.36 ± 7.70	**0.035**
SBP (mm of Hg)	135.28 ± 11.96	124.76 ± 9.83	**<0.01**
DBP (mm of Hg)	82.38 ± 8.92	78.80 ± 7.03	**0.028**
Hemoglobin (g/dl)	13.39 ± 1.21	12.86 ± 1.16	**0.029**
MCV (FL)	86.11 ± 5.18	87.29 ± 6.33	0.310
PCV (%)	40.56 ± 3.57	38.61 ± 3.56	**0.007**
WBC (ths/ul)	7.52 ± 1.25	7.14 ± 1.15	0.118
Platelets (ths/ul)	201.32 ± 46.77	209.62 ± 33.12	0.308
HBA1C (%)	8.50 ± 1.42	5.20 ± 0.22	**<0.01**
Blood Urea (mg/dl)	28.84 ± 6.46	27.12 ± 2.59	0.084
Serum Creatinine (mg/dl)	0.72 ± 0.16	0.74 ± 0.13	0.473
Serum Sodium (mmol/L)	138.90 ± 2.87	139.10 ± 1.72	0.674
Serum Potassium (mmol/L)	4.32 ± 0.42	4.07 ± 0.24	**<0.01**
Serum Bilirubin Total (mg/dl)	0.70 ± 0.20	0.71 ± 0.25	0.875
Serum Bilirubin Direct (mg/dl)	0.25 ± 0.08	0.20 ± 0.08	**0.005**
Serum Bilirubin Indirect (mg/dl)	0.45 ± 0.17	0.51 ± 0.23	0.176
Serum Albumin (g/dl)	4.29 ± 0.41	4.23 ± 0.27	0.364
Serum Globulin (g/dl)	3.17 ± 0.26	3.12 ± 0.33	0.451
Serum SGOT (U/L)	33.58 ± 10.9	37.6 ± 9.00	**0.047**
Serum SGPT (U/L)	43.32 ± 19.17	43.94 ± 16.77	0.864
Serum ALP (U/L)	103.72 ± 24.53	87.40 ± 20.44	**<0.01**
Total Cholesterol (mg/dl)	182.08 ± 52.75	161.56 ± 20.15	**0.012**
Triglycerides (mg/dl)	190.46 ± 75.50	185.66 ± 34.91	0.684
HDL-Cholesterol	45.96 ± 10.16	47.66 ± 6.24	0.316
LDL-Cholesterol	99.91 ± 36.41	89.74 ± 15.14	0.071
Vitamin-D	22.21 ± 8.97	20.63 ± 8.91	0.380

All the values are presented in mean ± standard deviation. p-value was significant at less than 0.05. Significant values are marked bold.

SBP, Systolic Blood Pressure; DBP, Diastolic Blood Pressure; PCV, Packed- cell volume; MCV, Mean corpuscular volume; WBC, White Blood Cell (count); SGOT, Serum Glutamate Oxaloacetate Transaminase; SGPT, Serum Glutamate Pyruvate Transaminase; ALP, Alkaline Phosphatase.

### 3.4 MASLD severity and liver assessment

**Figure 2:**
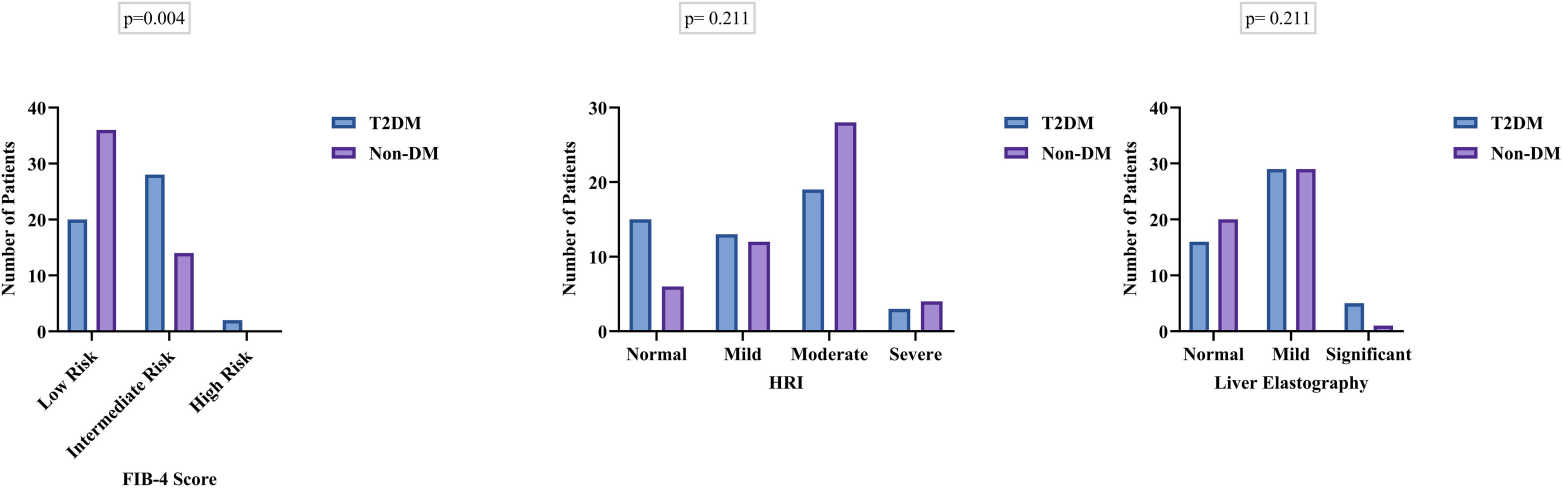
Comparison of MASLD indices (FIB-4, Hepato-Renal Index, and Liver Elastography) between T2DM and non-diabetic controls.

The distribution of fibrosis-4 (FIB-4) scores differed significantly between groups (p = 0.004). A greater proportion of T2DM patients were classified as intermediate-to-high risk (60%) compared with non-diabetic controls (28%), while the majority of non-diabetic participants (72%) fell into the low-risk category. Hepato-renal index (HRI) grades did not differ significantly between groups (p = 0.211). Moderate steatosis was the most frequent category in both groups, although it was more prevalent among non-diabetic controls (56% vs. 38%). Liver stiffness measurements obtained using two-dimensional shear wave elastography (2D-SWE) did not differ significantly between the groups (p = 0.211) (**Figure 2****).**

### 3.5 Correlation between clinical variables and bone mineral density

Patients in the T2DM group had significantly reduced lumbar spine (L1–L4) BMD and T-scores compared to controls (0.93 ± 0.13 vs. 0.98 ± 0.10; p = 0.026 and –1.33 ± 1.12 vs. –0.72 ± 0.90; p = 0.004), indicating a higher prevalence of osteopenia or osteoporosis among diabetic patients. At the left hip, BMD was comparable between groups (p = 0.218), but T-scores were significantly lower in the T2DM group (0.30 ± 0.88 vs. 0.73 ± 0.98; p = 0.024). No significant differences were observed at the left forearm for BMD or T-scores. These findings suggest that the coexistence of MASLD and T2DM may preferentially affect spinal bone mineral density, a clinically relevant site for fracture risk assessment (**Table 3****).**

**Table 3:** Comparison of Bone Mineral Density (BMD) and T-scores at lumbar spine (L1-L4), hip, and forearm between T2DM patients and non-diabetic controls.

Variables	T2DM (n=50)	Non- DM (n=50)	P-value
Lumbar vertebra (L1-L4)			
BMD (mean ± SD)	0.93 ± 0.13	0.98 ± 0.10	**0.026**
T-score (mean ± SD)	-1.33 ± 1.12	-0.72 ± 0.90	**0.004**
Left hip			
BMD (mean ± SD)	1.04 ± 0.13	1.08 ± 0.14	0.218
T-score (mean ± SD)	0.30 ± 0.88	0.73 ± 0.98	**0.024**
Left forearm			
BMD (mean ± SD)	0.60 ± 0.08	0.59 ± 0.07	0.718
T-score (mean ± SD)	-0.80 ± 1.27	-0.35 ± 1.20	0.071

Values are presented as mean ± standard deviation. A p-value less than 0.05 indicates statistical significance, and significant values are marked in bold.

Pearson correlation coefficients were calculated between age, BMI, BMD at lumbar spine, total hip, forearm, and liver indices (FIB-4, HRI, elastography). Age correlated positively with FIB-4 (r = 0.71, p < 0.001) and negatively with lumbar spine BMD (r = –0.23, p = 0.02), while BMI correlated positively with BMD at all sites (p < 0.05) (**Supplementary Table 1****).**

### 3.6 Multivariable regression analysis

Multivariable linear regression analyses were performed to evaluate associations between MASLD indices and bone mineral density at the lumbar spine (L1–L4), total hip (proximal femur), and forearm, adjusting for age, sex, and smoking status. No significant associations were observed between MASLD markers and lumbar spine or forearm bone mineral density. In contrast, a significant positive association was identified between the hepato-renal index and total hip bone mineral density (β = 0.738, p = 0.022), while fibrosis-4 and elastography values were not associated with bone mineral density. These findings indicate a site-specific relationship between hepatic steatosis and skeletal health, particularly at the hip (**Table 4****).**

**Table 4:** Multivariable linear regression of MASLD indices with bone mineral density T-scores at different skeletal sites.

Variable	B	95% CI (Lower–Upper)		P-value
Vertebral bone mineral density				
Age	-0.013	-0.039 to 0.014		0.343
Gender (Female)	-0.389	-0.831 to 0.054		0.084
Smoking	-0.290	-0.931 to 0.351		0.372
FIB-4 score	-0.413	-1.006 to 0.181		0.170
HRI	0.599	-0.071 to 1.269		0.079
Elastography value	0.012	-0.107 to 0.131		0.845
Total hip bone mineral density				
Age	0.002	-0.023 to 0.026		0.878
Gender (Female)	-0.152	-0.568 to 0.263		0.469
Smoking	-0.020	-0.623 to 0.583		0.949
FIB-4 score	-0.266	-0.823 to 0.292		0.347
HRI	0.738	0.107 to 1.368		**0.022**
Elastography value	0.047	-0.065 to 0.159		0.410
Left forearm bone mineral density				
Age	-0.027		-0.060 to 0.005	0.101
Gender (Female)	0.040		-0.512 to 0.592	0.886
Smoking	-0.270		-1.070 to 0.531	0.505
FIB-4 score	0.397		-0.344 to 1.138	0.290
HRI	0.524		-0.312 to 1.361	0.216
Elastography	0.043		-0.106 to 0.191	0.571

P-values less than 0.05 are considered significant, and significant values are marked in bold.

### 3.7 Subgroup analysis by age and sex

Subgroup analysis stratified by age and sex revealed site-specific differences in bone mineral density (**Table 5****).** Among males aged ≥50 years, lumbar spine (L1–L4) T-scores were significantly lower in the T2DM group compared with non-diabetic controls (–1.43 ± 1.25 vs. –0.35 ± 0.74; p = 0.024). Although hip and forearm T-scores were also lower among diabetic males, these differences did not reach statistical significance (p = 0.088 and p = 0.100, respectively).

**Table 5:** Subgroup analysis of T-score values at lumbar vertebra (L1-L4), hip, and forearm in males (≥50 years) and females (≥40 years) with T2DM compared to non-diabetic controls.

Variables	T2DM (n=22)	Non- DM (n=09)	P-value
Male ≥ 50years			
Lumbar vertebra (L1-L4)			
T-score (mean ± SD)	-1.43 ± 1.25	-0.35 ± 0.74	**0.024**
Left hip			
T-score (mean ± SD)	0.23 ± 0.85	0.76 ± 0.41	0.088
Left Forearm			
T-score (mean ± SD)	-0.84 ± 1.48	0.04 ± 0.74	0.100

Variables	T2DM (n=13)	Non- DM (n=12)	P-value
Female ≥ 40years			
Lumbar vertebra (L1-L4)			
T-score (mean ± SD)	-1.66 ± 1.09	-1.05 ± 1.09	0.177
Left hip			
T-score (mean ± SD)	0.33 ± 0.96	1.15 ± 1.11	0.060
Left forearm			
T-score (mean ± SD)	-0.93 ± 1.40	-0.41 ± 1.25	0.339

Values are presented as mean ± standard deviation. A p-value less than 0.05 indicates statistical significance, and significant values are marked in bold.

In females aged ≥40 years, T-scores at the lumbar spine, hip, and forearm were consistently lower in the T2DM group compared with controls; however, none of these differences were statistically significant (lumbar spine p = 0.177, hip p = 0.060, forearm p = 0.339).

## 4 Discussion

The present study investigated the relationship between Metabolic Dysfunction-Associated Steatotic Liver Disease (MASLD) and bone mineral density (BMD) in individuals with and without type 2 diabetes mellitus (T2DM) within a North Indian population. The findings reveal a significant association between MASLD and reduced BMD, particularly among T2DM patients, and highlight site-specific variations in skeletal involvement. Additionally, the study explored socio-demographic, clinical, and hepatic factors contributing to this complex interplay, offering important insights into the multisystemic nature of metabolic disease.

The study identified a higher prevalence of male participants and rural residence among T2DM individuals, consistent with prior South Asian data suggesting gender disparities in healthcare access and diagnosis (21). Although mean BMI was similar between groups, non-diabetics showed a higher proportion of Class II obesity, a trend possibly explained by the “obesity paradox” and sarcopenic obesity often seen in longstanding diabetes (22). Furthermore, smoking was more prevalent among diabetics, reinforcing its role as a modifiable risk factor for both insulin resistance and cardiovascular complications (23).

Biochemically, T2DM participants showed higher blood pressure, serum potassium, and total cholesterol, reflecting a broader metabolic syndrome profile. While vitamin D deficiency was prevalent across both groups, its ubiquitous nature in this cohort limited its interpretability as a distinguishing factor between diabetic and non-diabetic individuals (24).

In the present study, no significant differences were observed in overall liver stiffness or steatosis grades between T2DM patients and non-diabetic controls; however, advanced fibrosis (≥F2) was more prevalent among individuals with T2DM (10% vs. 2%). A significantly greater proportion of T2DM participants had elevated FIB-4 scores, suggesting a heightened risk of hepatic fibrosis. This is in line with established pathophysiological links between chronic hyperglycemia, insulin resistance, and fibrogenesis via hepatic stellate cell activation (25). Notably, hepatic steatosis (HRI) and liver stiffness (2D-SWE) did not differ significantly between groups, indicating that fibrosis progression may occur independently of steatosis quantity. This highlights the importance of using non-invasive fibrosis indices to detect subclinical hepatic involvement in T2DM patients (26).

A key finding of this study is the significantly reduced BMD and T-scores at the lumbar spine (L1-L4) in individuals with T2DM compared to non-diabetics, while at the hip and forearm remained comparable between groups. Among males aged 50 years and above, individuals with type 2 diabetes mellitus (T2DM) had significantly reduced bone mineral density (BMD) at the lumbar spine (L1-L4), compared to non-diabetic counterparts. The observed difference in lumbar spine (L1-L4) T-scores (–1.43 ± 1.25 vs. –0.35 ± 0.74; p = 0.024) suggests a site-specific susceptibility of trabecular bone to diabetic alterations. This finding is consistent with previous reports indicating that the axial skeleton, which is rich in trabecular bone, is more metabolically active and thus more vulnerable to the detrimental effects of chronic hyperglycemia and advanced glycation end products (AGEs) that impair bone collagen quality and microarchitecture (27, 28). Although hip and forearm T-scores were also lower in diabetic males, the differences were not statistically significant, which may reflect a relative preservation of cortical bone or limited sample size reducing statistical power. Interestingly, among females, T-score values across all measured sites were consistently lower in the T2DM group; however, none of these reached statistical significance. This trend may be influenced by multiple factors, including differences in hormonal status, smaller subgroup size, and the complex interplay between estrogen deficiency and diabetic bone disease (29).

These results support the hypothesis of site-specific bone loss, particularly affecting trabecular-rich skeletal sites. This aligns with prior research describing the paradox of normal or elevated BMD in T2DM but increased fracture risk, likely due to poor bone quality rather than quantity (30). Contributing mechanisms include advanced glycation end-product (AGE) accumulation, impaired osteoblast function, altered bone remodeling, and increased marrow adiposity.

Multivariable analyses revealed a significant positive association between hepatic steatosis (HRI) and hip BMD, and a borderline association with spine BMD, suggesting that early hepatic fat accumulation may correlate with preserved or increased BMD. These findings mirror those from prior population studies where mild MASLD was associated with higher BMD, potentially due to hyperinsulinemia and adipokine-mediated anabolic effects on bone (31, 32). However, this relationship is likely nonlinear; as MASLD progresses to fibrosis and systemic inflammation increases, bone quality may deteriorate despite stable or elevated BMD values.

In contrast, FIB-4 and liver stiffness measurements showed no significant association with BMD in this study, possibly reflecting the early-stage hepatic involvement in the cohort. This divergence emphasizes the need to distinguish between the effects of hepatic fat and fibrotic progression when assessing skeletal risk in MASLD.

The present study has several notable strengths. It specifically investigated site-specific changes in bone mineral density (BMD) in relation to MASLD among Indian patients with type 2 diabetes mellitus (T2DM), thereby addressing an important regional knowledge gap. A comprehensive approach was adopted for liver assessment, incorporating multiple non-invasive tools, including the FIB-4 score, hepato-renal index (HRI), and two-dimensional shear wave elastography (2D-SWE), which enhanced the robustness of MASLD evaluation. The study further strengthened its findings by adjusting for key potential confounders such as age, gender, and smoking in multivariable analyses. Additionally, detailed profiling of clinical, biochemical, and hematological parameters allowed for a thorough and contextual interpretation of the results.

However, this study has limitations. Its cross-sectional design limits causal inference between MASLD progression and skeletal alterations, and the single-center sample may restrict generalizability. Although dual-energy X-ray absorptiometry (DXA) remains the clinical standard for bone mineral density (BMD) assessment, it provides only areal bone estimates and does not capture critical determinants of bone strength, including microarchitecture, cortical porosity, trabecular connectivity, and material properties. Consequently, many individuals sustaining fragility fractures have normal or osteopenic DXA-derived BMD values, limiting individualized fracture risk prediction in MASLD-associated bone disease (33). Emerging evidence supports a hierarchical model of skeletal involvement in MASLD, wherein early alterations in bone quality and turnover precede measurable BMD loss, followed by microarchitectural deterioration and increased fracture susceptibility (34).

Recent advances aimed at improving fracture risk assessment in MASLD include high-resolution peripheral quantitative computed tomography, trabecular bone score, finite element–based biomechanical modeling, and integration of bone turnover markers with metabolic and inflammatory profiling, which better reflect the complex liver–bone crosstalk underlying skeletal fragility in MASLD (33, 34). The absence of bone quality assessments and fracture outcome data in the present study therefore limits translation of BMD findings into clinical fracture risk. Also, potential residual confounding from comorbidities, vascular disease, and sarcopenia, which were not systematically assessed, as well as the inherent limitations of the cross-sectional design. Bone turnover markers and histological assessment were beyond the scope of this study and are now highlighted as important areas for future research. Future longitudinal studies incorporating advanced imaging modalities, biochemical markers, and fracture endpoints are warranted.

## 5 Conclusion

This study found that MASLD is significantly associated with reduced BMD, particularly in individuals with type 2 diabetes mellitus, and that this association is site-specific, affecting predominantly trabecular-rich skeletal sites. Moreover, while early hepatic fat accumulation may correlate with increased BMD, progression toward fibrosis appears to attenuate this effect, potentially compromising bone quality. These findings underscore the importance of viewing MASLD as a multisystem metabolic disease with implications beyond liver health. Incorporating bone health assessments and hepatic fibrosis screening into standard diabetes care along with tailored lifestyle interventions, could help mitigate the long-term risks of osteoporotic fractures and liver-related complications in this growing patient population.

Future longitudinal and mechanistic studies are needed to further unravel the complex, nonlinear relationships between liver pathology, metabolic dysfunction, and skeletal health, particularly across diverse ethnic, gender, and age groups.

## Data Availability

The original contributions presented in the study are included in the article/**Supplementary Material**. Further inquiries can be directed to the corresponding authors.

## Supplementary material

Supplementary Table 1 Pearson Correlations Between Clinical Variables and BMD.
