# Supplementary material for: Metabolic dysfunction-associated steatotic liver disease and bone mineral density in type 2 diabetes mellitus: insights from a single-center cross-sectional study in North India
Source: Front Endocrinol (Lausanne). 2026 Mar 2;17:1758966. doi: 10.3389/fendo.2026.1758966 (PMC12989356; doi:10.3389/fendo.2026.1758966)
Supplement: Supplementary Table 1 — Pearson Correlations Between Clinical Variables and BMD. [file Table1.docx]

**Supplementary Table 1: Pearson Correlations Between Clinical Variables and BMD**

| **Variable** | **Age** | **BMI** | **Lumbar Spine BMD** | **Total Hip BMD** | **Left Forearm BMD** | **FIB-4** | **HRI** | **Elastography** |
| --- | --- | --- | --- | --- | --- | --- | --- | --- |
| **Age** | 1 | 0.08 (0.42) | –0.23* (0.02) | –0.07 (0.50) | –0.06 (0.54) | 0.71** (<0.001) | –0.12 (0.25) | 0.20 (0.05) |
| **BMI** | 0.08 (0.42) | 1 | 0.34** (<0.001) | 0.43** (<0.001) | 0.04 (0.67) | 0.01 (0.92) | 0.24* (0.02) | 0.21* (0.04) |
| **Lumbar Spine BMD** | –0.23* (0.02) | 0.34** (<0.001) | 1 | 0.63** (<0.001) | 0.55** (<0.001) | –0.20* (0.04) | 0.18 (0.08) | –0.02 (0.85) |
| **Total Hip BMD** | –0.07 (0.50) | 0.43** (<0.001) | 0.63** (<0.001) | 1 | 0.48** (<0.001) | –0.07 (0.47) | 0.24* (0.02) | 0.08  (0.45) |
| **Left Forearm BMD** | –0.06 (0.54) | 0.04 (0.67) | 0.55** (<0.001) | 0.48** (<0.001) | 1 | 0.07 (0.52) | 0.15 (0.13) | 0.04  (0.72) |
| **FIB-4** | 0.71** (<0.001) | 0.01 (0.92) | –0.20* (0.04) | –0.07 (0.47) | 0.07 (0.52) | 1 | 0.02 (0.84) | 0.08  (0.45) |
| **HRI** | –0.12 (0.25) | 0.24* (0.02) | 0.18 (0.08) | 0.24* (0.02) | 0.15 (0.13) | 0.02 (0.84) | 1 | –0.04  (0.70) |
| **Elastography** | 0.20 (0.05) | 0.21* (0.04) | –0.02 (0.85) | 0.08 (0.45) | 0.04 (0.72) | 0.08 (0.45) | –0.04 (0.70) | 1 |
| Pearson correlation coefficient (r) is shown with p-value in parentheses. *p < 0.05, **p < 0.01 (2-tailed).  BMD: Bone Mineral Density; BMI: Body Mass Index; FIB-4: Fibrosis-4 score; HRI: Hepato-Renal Index | | | | | | | | |
